# Effects of Treatment of Treadmill Combined with Electro-Acupuncture on Tibia Bone Mass and Substance PExpression of Rabbits with Sciatic Nerve Injury

**DOI:** 10.1371/journal.pone.0164652

**Published:** 2016-11-23

**Authors:** Yan Wang, Qiang Tang, Luwen Zhu, Ruyi Huang, Lei Huang, Melanie Koleini, Dequan Zou

**Affiliations:** 1 The 2nd affiliated hospital of Heilongjiang University of Chinese Medicine, Harbin, China; 2 Heilongjiang University of Chinese Medicine, Harbin, China; 3 HRPO, Washington University School of Medicine, St. Louis, Missouri, United States of America; 4 Program in Physical Therapy, Washington University School of Medicine, St. Louis, Missouri, United States of America; Federal University of Rio de Janeiro, BRAZIL

## Abstract

The peripheral nervous system may play an important role in normal bone maintenance and remodeling. Substance P (SP) is a neuropeptide associated with bone loss and formation that may mediate the effects of the nervous system. The purpose of this study is to determine if treadmill running combined with electro-acupuncture at Jiaji acupoints (Jiaji-EA) affects tibial bone mass and SP expression in rabbits with sciatic nerve injury. Twenty-four juvenile male New Zealand white rabbits were randomly assigned to one of 4 groups: sham injury control (sham), sciatic never crush control (SNCr), treadmill running (treadmill), and Jiaji-EA combined with treadmill running (ET group). The SNCr, treadmill, and ET groups all had an induced sciatic never crush injury of approximately 2mm. Control groups received no intervention; the treadmill and ET groups were trained by treadmill; the ET group also received Jiaji-EA. After the 4 weeks of treatment, toe-spreading index (TSI), BMD, bone strength, and SP expression in the tibia were significantly lower in the nerve injury groups (SNCr, treadmill, and ET) compared to the sham groups (*p*<0.05). Treatment (treadmill and ET groups) increased all measures compared to the SNCr group (*p*<0.05). Further, TSI, BMD, bone strength, and SP expression in the ET group were higher than the treadmill group (*p*<0.05). Our results indicate that treadmill therapy combined with electro-acupuncture at Jiaji acupoints prevents bone loss in rabbit tibias after sciatic nerve injury. This may occur in two ways: indirectly in association with axon regeneration and directly via loading on the bone mediated through increased SP expression. This study provides important evidence for the clinical treatment of bone loss after peripheral nerve injury.

## Introduction

Injuries to peripheral nerves differ from injuries to most other tissue types because further neuron and target organ degeneration often follows [1]. Fortunately, the peripheral nervous system (PNS) has an innate capacity to regrow to their targets, including muscle, bone, and skin. However, the regeneration process is slow and incomplete [2] and is often accompanied by disturbing motor, autonomic and sensory consequences [3]. The peripheral nervous system may play an important role in bone healing following fracture or other trauma [4–6]. Bone fractures accompanied by peripheral nerve injuries heal slower [7] and sensory denervation negatively affects long-term prognosis[8]. Substance P (SP), a neuropeptide belonging to the tachykinin family, is widely distributed in the body, particularly in the central and peripheral nervous systems where it acts as a neurotransmitter or neuromodulator [9]. Studies suggest that the absence of SP reduces bone formation rate associated with fracture healing [10,11].

Exercise affects bones in both humans and animals. Dynamic exercise is superior to static exercise in increasing bone mass and greater intensity training leads to greater increases in bone mass [12]. Increased mechanical loading is also beneficial to bone growth. Running-generated impact and load on bone are believed to increase cortical bone mass through remodeling.

Acupuncture, a therapy originating in China, has been widely used for the treatment of neurological disorders, including spinal cord injury (SCI) [13]. Electro-acupuncture (EA) at the acupoints of Governor Vessel (Du Meridian) and Jiaji has proven to have a therapeutic effect in the treatment of spinal cord injury (SCI) both in clinical care and animal experiments [14]. Jiaji acupoints are located on the bilateral spinous processes of lumbar spine [15], 5mm lateral to the posterior midline in rabbits. The sciatic nerve and lumbosacral plexus are located beneath these acupoints. Some studies [16] suggest the sciatic nerve of the rabbit is located between L_7_ and S_2_ in the spinal cord. However, due to the relationship between sections of lumbosacral spinal cord and their lumbosacral vertebras in rabbit, we believe that the sciatic nerve is located between L_4_ and L_6_ of the of lumbosacral vertebras. Neurons of the sciatic nerve and lumbosacral plexus are beneath these acupoints. Since adequate connectivity in spinal circuits and peripheral nervous system integration are also important factors in nerve regrow [17], Jiaji-EA may help to facilitate sciatic nerve regeneration after the nerve injury via stimulation of the sciatic nerve and lumbosacral plexus.

In our previous animal experiments and clinical practices, we observed that bones that were denervated due to peripheral injury were apt to suffer bone loss followed by pathological fracture. Previous studies focused primarily on the damaged peripheral nerves themselves and denervated muscle [18,19]. Although bone healing combined with peripheral nerve injury has been investigated [4,5,7,8], interventions for denervated bones have not been extensively studied. This study measures the effects of combining treadmill running with Jiaji-EA on tibia bone mass, bone strength and SP expression in rabbits with sciatic nerve injury. We hypothesize that treadmill running combined with Jiaji-EA will decrease bone loss and weakening, and increase SP expression, reducing pathological fracture of denervated bones.

## Materials and Methods

This study was carried out in strict accordance with the recommendations in the Guide for the Care and Use of Laboratory Animals of Chinese Academy of Medical Sciences. The protocol was approved by the Committee on the Ethics of Animal Experiments of the Second Affiliated Hospital of Heilongjiang University of Chinese Medicine, China (Permit Number: ABX20140105A). All surgery was performed under sodium pentobarbital anesthesia, and all efforts were made to minimize suffering.

### Subjects

Twenty-four 11–12 weeks-old male New Zealand white rabbits with a mean weight of 1.78±0.12 kg were randomly assigned to one of 4 groups: sham (n = 6), SNCr (n = 6), treadmill (n = 6), or Jiaji-EA combined with treadmill (ET, n = 6). All animals were anesthetized with 1.5±0.5ml/kg of 10% chloral hydrate via the ear marginal vein. In the SNCr, treadmill, and ET groups, a lateral approach was created on the left hind limb. The incision was located mid-way between the attachment point of rectus femoris tendon on knee joint and the rectus femoris between the upper one third and the midline. The tip of hemostatic forceps covered by thin plastic tubing were used to crush the nerve trunk by full clamping the nerve for 5 minutes [20,21]; this caused damage across approximately 2mm of the nerve. The sham group was subjected to the same surgical procedure with sciatic nerve exposure but without crush injury.

### Intervention Groups

**Fig 1:**
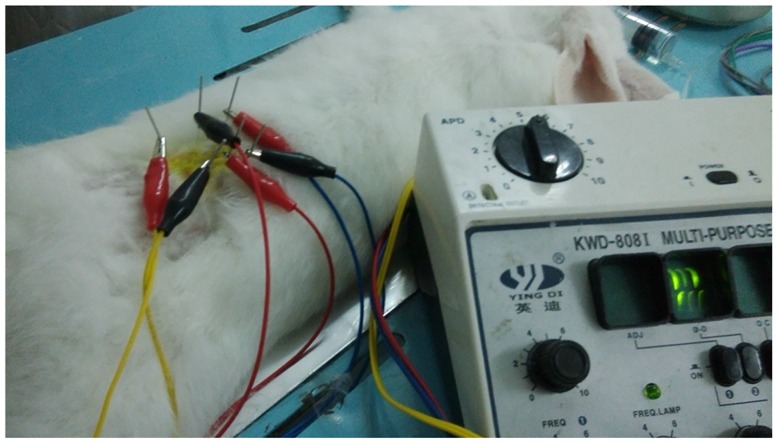
The therapy of Electro-acupuncture. The four limbs of each rabbit in the ET group were fixed on the experimental table in the prone position by a thick cotton band. Six sterile needles were inserted into each acupoint to a depth of 10-15mm. Then the needles were connected to a 3Hz/s electrical current.

The intervention methods for all groups were as follows: (1) Control groups (sham and SNCr): no intervention, rabbits were removed from cage and placed on a treadmill while treadmill was not operating for 20min each day, as a sham procedure; (2) Treadmill group: before surgery, the rabbits were made to adaptively run on a treadmill on 3 consecutive days. Three days post-surgery, the animals were made to run for 20 minutes a day at a rate of 10m/min for 3 days, at a rate of 15m/min for 20 min/day from the fourth to sixth day, and then at a rate of 20m/min for 20 min/day from the seventh day onward, 6 days/week for a total treatment cycle duration of 4 weeks; (3) ET group: rabbits received treadmill exercise using the same protocol as the treadmill group. In addition, 3 days post-surgery, 6 Jiaji acupoints (3 acupoints on each side) were identified bilateral of the spinous processes of L_4_ to L_6_ of spine approximately 5mm lateral to the posterior midline. The length of the Jiaji region is approximate 30mm. The four limbs of each animal in the ET group were fixed on the experimental table in the prone position by a thick cotton band. The fur in the acupoint region was shaved and sterilized using iodophor, then a sterile needle (0.20mm in diameter and 25mm in length) was inserted into each acupoint to a depth of 10-15mm. Then the needles were connected to a 3Hz/s electrical current for 30 min/day, 6 days/week for a total treatment duration of 4 weeks (Fig 1). The rabbits in SNCr and Treadmill groups were given a sham procedure, with shaving of back and confinement but without needle insertion and application of electrical current. No anesthesia was used during the electro-acupuncture procedure and the rabbits did not display any increased pain behaviors.

After 4 weeks of treatment, all animals were anesthetized with 1.5±0.5ml/kg of 10% chloralhydrate via ear marginal vein and their hearts were cut to bleed out after the animals became deeply unconscious to euthanize them. Their left tibias were harvested by disarticulation at the knee and ankle. First, all ligaments around the knee joint and the tendons of gastrocnemius attached at medial and lateral condyles of femur were severed and the tibia was dislocated from the femur. The Achilles tendon was then cut. Next, the entire tricipital muscle was excised from the soft tissues connected to the tibia. Then the anterior tibial muscle was removed from tibia. The ligaments connecting the ankle joint were sheared, separating the tibia and foot. Finally, of the remaining soft tissue on the tibia was removed.

#### (1) Functional Analysis (FA)

The motor function of the left hind limb was assessed before the crush injury and 4 weeks post injury using the toe-spreading reflex. The toe-spreading reflex is a reliability, sensitivity and non-invasive method for assessing recovery of peroneal nerve function after injury[22,23]. The procedure was as follows: each rabbit was held by the loose skin of its back then was suddenly lowered through the air without letting them contact a surface. Rabbits with functional nerve innervation reflexively spread their second, third and fourth toes. The movement of these toes was captured using a digital video camera (I-phone 5, USA) and then was graded following the toe-spreading index (TSI) described by E. Gutmann (Table 1)[22,23].

**Table 1:** The toe-spreading index for behavioral analyses.

Grade	Symptoms
**1**	Just visible spreading of the 4th toe alone (also 2nd and 3rd)
**2**	Slight spreading of all three toes
**3**	Spreading of all three toes (less forceful than normal)
**4**	Full spreading of all three toes (equal to normal)

#### (2) Bone strength

Tibias were wrapped in saline soaked gauze and frozen (-20°C)after excision. Bone strength was measured by 3-point bending testing using a Universal Testing Machine (Zwick Z010, Germany) the same day. The tibia was put on the testing apparatus with anterior aspect up. The 3-point bending test was performed with a support span of 60 mm and strain rate of 1mm/min. The Ultimate Load of the tibia was measured at bone failure [24].

#### (3) Bone Mineral Density (BMD)

The bone mineral density of each tibia was measured using Dual-Energy X-ray absorptiometry (DXA) (Noland, USA). After strength testing, the bone was divided into two sections. The proximal section was placed on white paper in horizontal position with anterior side up under the DXA. A rectangular area of 1.53cm^2^ approximately 1cm below the tibial plateau was scanned to measure BMD.

#### (4) SP expression

After bone strength testing, a 10mm think sample of cortical bone from approximately the middle of the tibial shaft was sectioned perpendicular to the long axis of each bone (approximate cylinder) and fixed in a formaldehyde solution. A second bone sample was used for BMD testing. The bones were demineralized using EDTA-NaOH. Forty to 50 g of EDTA⋅2Na was added to 500ml of distilled water and 0.2g of NaOH. Another 350ml of distilled water was added and mixed, and then additional water was added to bring the final volume up to 1000ml. The bone tissue specimens were put into the solution and placed in a 4°C refrigerator and checked daily. The solution was replaced every second day. Bones were considered demineralized when the tissue specimens could be easily stabbed by a pin. Once demineralized, the tissue specimens were washed with distilled water and then embedded in paraffin. Five-micron thick sections of bone (5 sections for each rabbit) were cut using immunohistochemistry method for SP expression. After deparaffinization, the sections were submerged in a solution of phosphate-buffered saline (PBS) with 0.3% Triton-X 100 (pH 7.4) for 30 min and a solution of 0.3% H2O2for 1 h in order to block endogenous peroxidase. The sections were then incubated with polyclonal SP rabbit antiratantibody diluted 1:2000 in a solution consisting of1% bovine serum albumin and 0.05% sodium azide in0.1 M PBS for 24 h at 4°C. After three washings in PBS, the specimens were then exposed to biotinylated goat antirabbitor -mouse IgG diluted 1:200 in PBS for 4 h at room temperature. Three washings in PBS were followed by treatment with ABC diluted 1:100 in PBS for4 h at room temperature. The peroxidase reaction was then developed for 10 min in 0.05 M TRIS buffer (pH 7.6) containing 0.02% 3,3-diaminobenzidine tetrahydrochloride and 0.006% H2O2. [25]. The density of SP-positive staining was analyzed using a Digital medical image analysis system (Motic Med 6.0) [26].

### Analysis

Statistical calculations were performed using SPSS 19.0 for Windows. All data are presented as means ± SD. All variables were tested for normal distribution using the Kolmogorov-Smirnov test (*p*> 0.05). One-way analysis of variance (ANOVA) followed by the LSD post-hoc test was used for the statistical analysis. A significant difference was defined as *p* ≤ 0.05.

## Results

### 1. Functional Analysis (FA)

**Fig 2:**
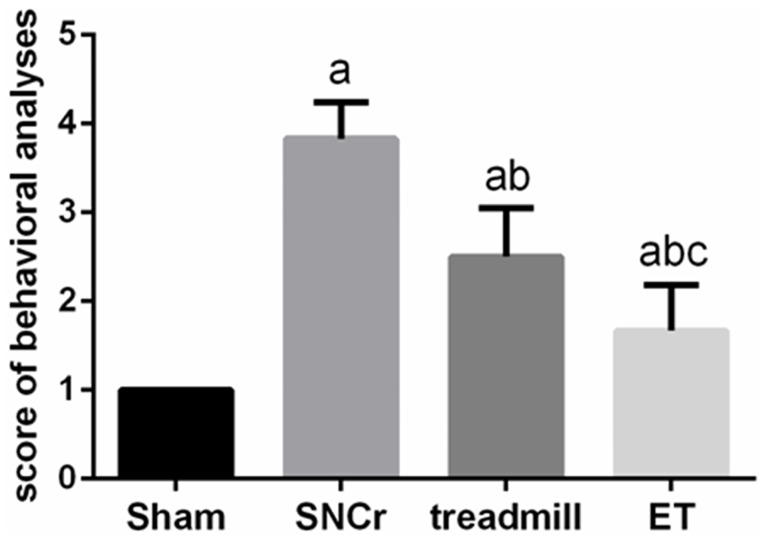
Comparison of toe-spreading index for behavioral analyses. After 4 weeks of treatment, compared with Sham group, ^a^*P*<0.05; Compared with SNCr group, ^b^*P*<0.05; Compared with treadmill group, ^c^*P*<0.05.

Motor function recovery was assessed by TSI at 4 weeks of treatment. Before the crush injury, all of TSI measures were grade 4. After 4 weeks of treatment, TSI in injury groups (SNCr, treadmill and ET) were reduced compared to the sham group (*p*<0.05), The TSI in the treadmill and ET groups was significantly higher compared to the SNCr (*p*<0.05). Further, the TSI in the ET groups was significantly higher than the treadmill group (*p*<0.05) (Fig 2).

### 2. BMD of the tibia

Injury to the sciatic nerve led to significant reductions in the BMD of the rabbits’ tibias. The SNCr, treadmill, and ET groups all had decreased BMD compared to the sham group (*p*<0.05). The BMD of the tibia in the treadmill and ET groups was significantly higher compared to the SNCr (*p*<0.05). Further, the BMD of the tibias in the ET group was significantly higher than the treadmill group (*p*<0.05) (Table 2).

**Table 2:** Comparison of BMD, bone strength and SP 4weeks post treatments.

Group(n = 6)	BMD (g/cm^2^)	Bone strength (N)	SP
Sham	0.197±0.008^a^	199.043±16.123	0.035±0.004
SNCr	0.123±0.008	51.315±11.459 ^a^	0.010±0.005 ^a^
Treadmill	0.151±0.002^a^^,^^b^	86.507±20.942^a^^,^^b^	0.017±0.001^a^^,^^b^
ET	0.176±0.005^a^^,^^b^^,^^c^	158.539±41.292^a^^,^^b^^,^^c^	0.025±0.002^a^^,^^b^^,^^c^

^a^ p<0.05 vs. sham group;

^b^ p<0.05vs.SNCrl group;

^c^ p<0.05vs.treadmill group.

### 3. Bone strength of the tibia

Significant reductions in the bone strength of the left tibia were noted in the SNCr, treadmill, and ET groups compared to the sham group (*p*<0.05). Bone strength in the treadmill and ET groups was significantly higher than the SNCr group (*p*<0.05). The bone strength in the ET group was significantly higher than the treadmill group (*p*<0.05)(Table 2).

### 4. SP expression in the tibia

**Fig 3:**
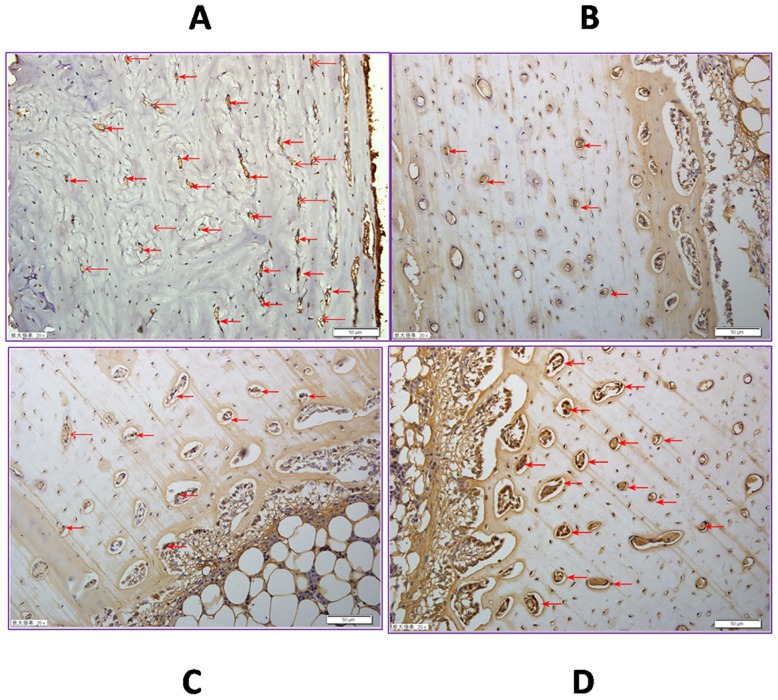
Substance P semi quantitation of left tibia 4 weeks post treatment. SP staining is shown in brown (red arrow). Scale bar = 50μm. Values are means ±SD of six rabbits in each group. **A**. SP in sham groups were higher than all others (*p*<0.05); **B**. SP in SNCr group was less than all others (*p*<0.05); **C**.SP in treadmill group was higher than SNCr group (*p*<0.05), less than ET group (*p*<0.05); **D**. SP in ET group was higher than other groups (*p*<0.05), except sham groups, was lower than sham groups (*p*<0.05).

Significant reductions in the SP expression in the tibia were found in the SNCr, treadmill, and the ET groups compared to the sham group (*p*<0.05). SP expression was partially restored by treadmill running. Both the treadmill and ET groups were significantly higher compared to the SNCr group (*p*<0.05). SP expression in the ET group was significantly higher compared to the treadmill group (*p*<0.05) (Table 2 and Fig 3).

## Discussion

The purpose of this study was to determine if dynamic, weight-baring exercise combined with Jiaji-EA improves tibial bone health in rabbits with sciatic nerve injury. In this study, we found that TSI, BMD, bone strength, and SP expression in the tibia are reduced after sciatic nerves injury. TSI,BMD, bone strength, and SP expression can be partially restored by intervening with treadmill running alone. However, TSI, BMD, bone strength, and SP expression levels were further improved by added Jiaji-EA to the treadmill therapy. In the current study, the better TSI,BMD and bone strength found in the ET group are associated with increased expression of SP. SP—immunoreactive nerve fibers innervate the bone and adjacent tissues [27]. SP-immunoreactive axons have been localized in bone, and SP receptors are widely distributed in osteoclasts and osteoblasts [28].

The status of innervation is important for bone union and functional recovery [29]. The lack of neural control may delay fracture healing [30]. Consistent with earlier research, we observed that the BMD and bone strength of the tibia are reduced after sciatic nerves injury[4.5.7.11.29]. Post-traumatic bone loss may be partially the result of decreased functional load bearing (disuse osteoporosis) [31]. This osteopenia can lead to osteoporosis and increased fracture risk [32]. Bone tissues are physiologically exposed to mechanical loading through physical activity including exercise, which contributes to development of growing bone [33,34]. In the current study, exercise training(treadmill running) increased cortical thickness in growing bone [35].

Accordingly, load bearing, active exercises are predicted to be among the most important methods to prevent bone loss and to help maintain BMD [36,37]. Various animal models and training modes, including treadmill running, jumping, free fall landing, and passive resistant training, indicate regular exercise is beneficial to bone development and health [37–41]. In this study, we chose treadmill running as the exercise intervention because it is active, full loading, and can be quantified. The main effects of treadmill running may be due to the suppression of bone mass reduction [38]. BMD and bone strength of tibia in the treadmill group were significantly higher compared to the SNCr group but still significantly lower than the sham control, indicating decreases in load baring play a part but are not solely responsible for bone loss. Running-generated impact and load on bone may suppress metabolic turnover and increase cortical bone mass through increased periosteal apposition [42]. Our results demonstrate that treadmill running can effectively preserve bone mass, consistent with previous findings [43–46].

Several neuropeptides may be local modulators of bone metabolism, influencing periosteal and medullary blood flow, angiogenesis, and nociception, in addition to having direct effects on osteoblasts and osteoclasts [4,47]. Furthermore, a recent study showed that bone and periosteum are innervated by sympathetic and sensory nerve fibers, implicating the peripheral nervous system in bone metabolism and indicating sensory and sympathetic neurotransmitters have crucial trophic effects essential for proper bone formation [11]. Studies of nerve regeneration in animals use a variety of electrophysiological and morphological tests, but these endpoints do not necessarily correlate with the return of muscle function [23]. Alternatively, FA value can be used to directly determine the prognosis of motor function after peripheral nerve injury [48]. TSI is believed to be an assessment of function that assesses the endpoint of peroneal nerve regeneration [23]. We found that the TSIs of rabbits in SNCr group improved little in the 4 weeks after injury, similar to a previous study [48]. However, the TSIs of rabbits in ET group were superior to all other injury groups. This indicates using Jiaji-EA combine with treadmill running is beneficial for sciatic never regeneration after injury.

Nerve regeneration plays a crucial role in improving bone mass. Changes at the spinal cord level may persist for a long time, contributing to chronic deficits after peripheral nerve injuries [49,50]. Thus, modulating the plastic changes at the spinal cord level are important to improve functional recovery [51]. Since the effectiveness of electro-acupuncture at Jiaji acupoints treatment of SCI has been demonstrated in both humans and animals[14], we believed that electrical stimulation of the Jiaji acupoints between L_4_ and L_6_ of the spine stimulates the sciatic nerve neurons. In addition, our previous study shows that secondary degeneration of neurons is reduced and axon regeneration is facilitated by Jiaji-EA after peripheral nerve injury [unpublished data]. In this study, we observed that the TSI, BMD, bone strength, and SP of tibia in the ET group were significantly increased compared to the SNCr and treadmill groups. These results suggest that bone metabolism may be improved by Jiaji-EA combined with treadmill indirectly in association with axon regeneration and directly via loading on the bone.

Substance P-immunoreactive nerve fibers innervate the bone and adjacent tissues. SP frequently co-localizes with calcitonin gene-related peptide in the peripheral sensory axons of bones [27]. The axon density varies depending on the stage of skeletal development, the location in the body and pathological conditions [18], suggesting a relationship between neuropeptides and bone. In this study, we found significant reductions in the SP expression in the tibia in the SNCr, treadmill, and ET groups compared to the sham group. Our data is consistent with the findings of Kingery al et [52]. They observed rapid, widespread loss of trabecular bone mass in both hind limbs after unilateral sciatic nerve transection in rats. They purposed that the contralateral neurogenic extravasation response was diminished and there was a contralateral reduction in bone SP expression after nerve injury, suggesting inhibiting SP signaling has negative effects on bone mass [52]. Other studies report that SP stimulates osteogenesis and the late stage osteoblastic bone formation [53,54] and the absence of SP reduces the rate of bone formation [30]. Grässel believes that during endochondral ossification, sensory neuropeptide SP promotes proliferation of stem cells and growth plate chondrocytes [55]. In this study, after sciatic nerve injury, tibial SP expression was significantly higher in the treadmill treatment group and highest in the group that received both treadmill and Jiaji-EA therapy. BMD and bone strength were positively correlated with SP expression. The bone health of the juvenile rabbits’ tibias was improved by treadmill running. Adding Jiaji- EAto the therapy produced increased benefits.

## Limitations

The current findings point to better recovery in the ET group, however, these results are limited by having no way to determine if the current results were due specifically to stimulation of the Jiaji-EA or the eletrostimularion of the general region. Further studies will be needed to determine this. Another limitation is the absence of direct evidence regarding the path by which treadmill combined with Jiaj-EA increased SP expression and improved bone mass after peripheral nerve injury. Further studies will be required to identify the mechanism by which treadmill combined with Jiaji-EA reverses denervation bone loss via signaling pathways.

## Conclusion

The TSI, BMD, bone strength, and SP expression of the tibia decrease after sciatic nerves injury in rabbits. The TSI, BMD and bone strength values were higher in treadmill and ET groups than in SNCr only group. The therapy of treadmill running combined with Jiaji-EA is superior to treadmill running only. Bone mass of rabbits’ tibias after sciatic nerve injury was increased by treadmill running combined with Jiaji-EA which is related to increasing the SP expression in the tibia. Combined treatment may facilitate bone mass retention in two ways: indirectly by facilitating axon regeneration and directly via loading on the bone.

## Supporting Information

S1 Table The details of BMD, Bone strenth, SP and FA. (XLSX)
